# The efficacy of vitamin D supplementation on painful diabetic neuropathy: Protocol for a systematic review and meta-analysis

**DOI:** 10.1097/MD.0000000000020871

**Published:** 2020-07-31

**Authors:** Wenjing Wei, Yanli Zhang, Rumeng Chen, Xianliang Qiu, Yang Gao, Qiu Chen

**Affiliations:** a School of Clinical Medicine; b Department of Endocrinology, Hospital of Chengdu University of Traditional Chinese Medicine, Chengdu, P.R. China.

**Keywords:** meta-analysis, painful diabetic neuropathy, systematic review, vitamin D

## Abstract

**Background:**

Painful diabetic neuropathy (PDN) is one of the main and severe complications of diabetic patients, which not only accelerates the occurrence of ulcers of diabetic foot and amputation of lower extremities but also severely affects the quality of life. It is common that vitamin D deficiency in diabetic patients and especially in these patients diagnosed with diabetic peripheral neuropathy. Previous studies have proved that there is an apparent vitamin D deficiency in PDN patients, and vitamin D supplementation can effectively improve patients’ pain symptoms and neurologic function. However, the evidence of these studies is unconvincing. Therefore, our research objective is to explore the effectiveness and security of vitamin D supplements on PDN.

**Methods:**

We will include randomized controlled trials on vitamin D supplementation in the treatment of PDN. And we will retrieve 8 electronic databases concerning this theme. The English databases mainly retrieve PubMed, Web of Science, Embase, and the Cochrane Library, while CNKI, VIP, CBM, and Wanfang database will be used to retrieve the Chinese Literature. There is no definite time limit for retrieval literatures, and the languages will be limited to Chinese and English. Besides, some clinical registration tests and gray literatures are also researched by us. The primary outcomes of our study are the amelioration of pain symptoms and assessment of peripheral nerve function. And some changes of biochemical indicators including fasting blood glucose, 2 hours postprandial blood glucose, glycosylated hemoglobin, calcium, and serum vitamin D level from preintervention and postintervention, adverse events will be regarded as secondary outcomes. The Review Manager RevMan5.3 will be used for meta-analysis of studies are included.

**Results:**

In this systematic review and meta-analysis, higher quality data evidence on vitamin D supplementation for PDN will be provided.

**Conclusion:**

Our study will eventually provide a proof of the efficacy and safety of vitamin D supplementation in patients with PDN, and to add a new option for the prevention and treatment of PDN patients.

**INPLASY registration number:**

INPLASY202050065

## 1 Introduction

Painful diabetic neuropathy (PDN), a syndrome of nerve disorders caused by diabetes mellitus, is one of the most common of the diabetic peripheral neuropathy (DPN). And as the main pathogenic factor of diabetic foot, PDN may cause ulcers in patients with diabetic foot and even irreversible amputation in the late stage.^[1]^ The main clinical features of PDN is varying degrees of pain symptoms with nocturnal exacerbation, and severe sleep disorder, and a considerable decline in the quality of life.^[2]^ According to relevant epidemiologic reports, the incidence of pain symptoms in patients with diabetes is 10% to 20%, while in patients with neuropathy is as high as 40% to 50%.^[3]^ the degree of pain of DPN is mainly divided into mild, moderate and severe, and the clinical manifestations are different. Compared with painless diabetic neuropathy, PDN plays a more destructive role in the psychosomatic health of diabetic patients.^[2]^ As one of the most devastating and crippling complications of diabetes, on the one hand, PDN can lead to a decline in the quality of daily life and an raising in mortality. On the other hand, it can result in a huge medical burden.^[4]^ In America, the total medical cost of diabetics is $6632 a year, while PDN patients with severe pain spend about four times as much as diabetics.^[2]^ Hence, early detection of high-risk factors of PDN is of great significance in preventing foot ulcers and reducing the incidence of limb amputation.^[5]^

At present, experts and clinicians agree that the analgesic effect of drug treatment of neuropathic pain has nothing to do with the etiology of neuropathy.^[6]^ All kinds of symptomatic analgesic drugs including tricyclic antidepressants, selective serotonin-norepinephrine reuptake inhibitors, selective serotonin reuptake inhibitors, anticonvulsants, and opioids are all used to relieve symptoms.^[7]^ However, the effective treatment of PDN is far from satisfactory,^[8]^ with existing the symptomatic treatment of western medicine only providing limited pain relief, often with significant side effects that the patient cannot tolerate. Alternative therapies have been seemed as a new option for DPN treatment, because of the advantage of having few side effects and excellent curative effects. Therefore, alleviating PDN to improve the quality of daily life has become a major clinical research problem. PDN should be considered as a neuropathy syndrome that is clinically different from DPN, and we will face significant challenges in patient management of PDN.^[9]^

It is clear that serum vitamin levels are lower in DPN patients, but there are few reports of PDN patients. Compared with DPN patients without pain, vitamin D has a significant influence on PDN.^[10]^ The clinical trials of Basit et al^[11]^ have shown that high-dose intramuscular injection of vitamin D can effectively relieve the pain symptoms of patients. There are also show that the level of vitamins in DPN is decreased and oral vitamin D supplementation improved vitamin D status and the peripheral nerve function.^[12]^ Razzaghi et al^[13]^ studies have shown that vitamin D supplementation is conducive to wound healing of diabetic foot ulcers and the metabolic state of diabetic patients. In conclusion, there are some lack of understanding of vitamin D is an important factor in neuropathic pain in PDN. And it is possible that vitamin D supplementation is an effective “analgesic” in mitigating pain in patients with DPN.^[14]^ However, based on the current evidence of randomized controlled trials on vitamin D in the treatment of DPN published in public is insufficient, and relevant systematic review and meta-analysis is rare.

## 2 Methods

### 2.1 Protocol registration

According to the guidelines of the Preferred Reporting Items for Systematic Reviews and Meta-analysis Protocol (PRISMA-P),^[15]^ our protocol has been registered on the website of International platform of registered systematic review and meta-analysis protocols of INPLASY. The registration number is INPLASY202050065 DOI:10.37766/inplasy2020.5.0065. We will report any adjustments that may occur during the study in the final report.

### 2.2 Inclusion criteria

#### 2.2.1 Type of study

Only randomized controlled trials of vitamin D supplementation on PDN can be included. There is no time limit for publication and language is restricted to Chinese and English in the initial search. However, nonrandomized controlled trials, animal experiments, reviews, and case reports are excluded.

#### 2.2.2 Type of participant

Participants who meet the following 3 criteria will be included:

1. Diabetic patients under 75 years old, whether patients with type 1 or type 2 diabetes
2. Diagnosis of DPN
3. Have symptoms of pain and symmetrical pain in the distal lower extremities lasted at least 3 weeks

#### 2.2.3 Type of intervention

No matter the experimental group or the control group, they are also cured with routine essential treatment of diabetes, including diet, exercise or drug therapy to control the level of blood glucose. Besides, all kinds of symptomatic analgesic drugs also are used to relieve symptoms. On this basis, vitamin D supplementation on PDN is used for experimental group, without limitation about dosage and form of vitamin D, while the control group is limited to basic treatment. In addition, neither group took any drugs that affect the outcome indicators.

#### 2.2.4 Types of outcome measurements

##### 2.2.4.1 Primary outcome

The primary outcomes concerning the improvement of pain symptoms and assessment of peripheral nerve function. The degree of improvement of pain symptoms is measured by different pain score scales. Peripheral nerve function includes the score of neurologic symptoms and the examination of nerve conduction function. All symptom survey scores are reported by patients.

##### 2.2.4.2 Secondary outcomes

Some changes of biochemical indicators including fasting blood glucose, 2 hours postprandial blood glucose, glycosylated hemoglobin, calcium and serum vitamin D level are included in secondary outcomes. Besides, adverse events in the study are also included. The difference from preintervention to postintervention will be recorded.

#### 2.2.5 Exclusion criteria

Participants who meet one or more of these items will be excluded:

1. Glycosylated hemoglobin > 11%
2. There is a diagnosis of diabetic foot and foot ulcers occur
3. Neuropathic pain is not caused by PDN (e.g., malignant tumors, vitamin B_12_ deficiency, hypothyroidism, neurotoxic drug therapy, etc)
4. Diabetic patients with serious acute and chronic complications, such as diabetic ketoacidosis, etc
5. Oral and injection of vitamin D and calcium or multivitamins in the past 3 months
6. Additionally, nonrandomized controlled trials, animal experiments, reviews, observational studies, retrospective studies, and case reports are excluded

### 2.3 Search methods

#### 2.3.1 Electronic data sources

There are 8 databases will be searched. The English databases mainly retrieve PubMed, Web of Science, Embase, and the Cochrane Library, while CNKI, VIP, CBM, and Wanfang database will be used to retrieve the Chinese literature.

#### 2.3.2 Other resources

To retrieve as many literatures as possible that meet the inclusion criteria, apart from the above 8 electronic databases, we also collect information through other channels, such as the clinical trial registries and gray literature.

### 2.4 Search strategy

A commonly used retrieval strategy, the integration of MeSH subject words and free-text Words, is adopted by all the reviewers, MeSH headings: Diabetic Neuropathies, Vitamin D and Randomized controlled trial, Broad free terms: Diabetic Neuropathy, Neuropathies, DiabeticNeuropathy, DiabeticNeuralgia, DiabeticNeuralgiasPainful; Diabetic Neuropathies, Painful; Neuropathies, Painful; vitamin d; 25 (OH)D; 25-HydroxyvitaminD; hypovitaminosisD; ControlledClinicalTrial, randomized, placebo. The search strategy for PubMed is summarized in Table 1 as an example. Of course, there is an appropriate judgment in line with the actual search situation. Other electronic databases will be searched using the similar strategy.

**Table 1:**
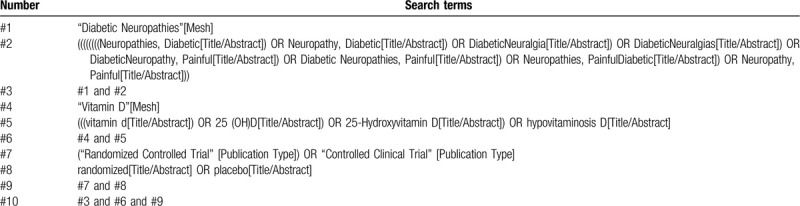
Example of PubMed search strategy.

### 2.5 Data extraction

#### 2.5.1 Selection of studies

**Figure 1:**
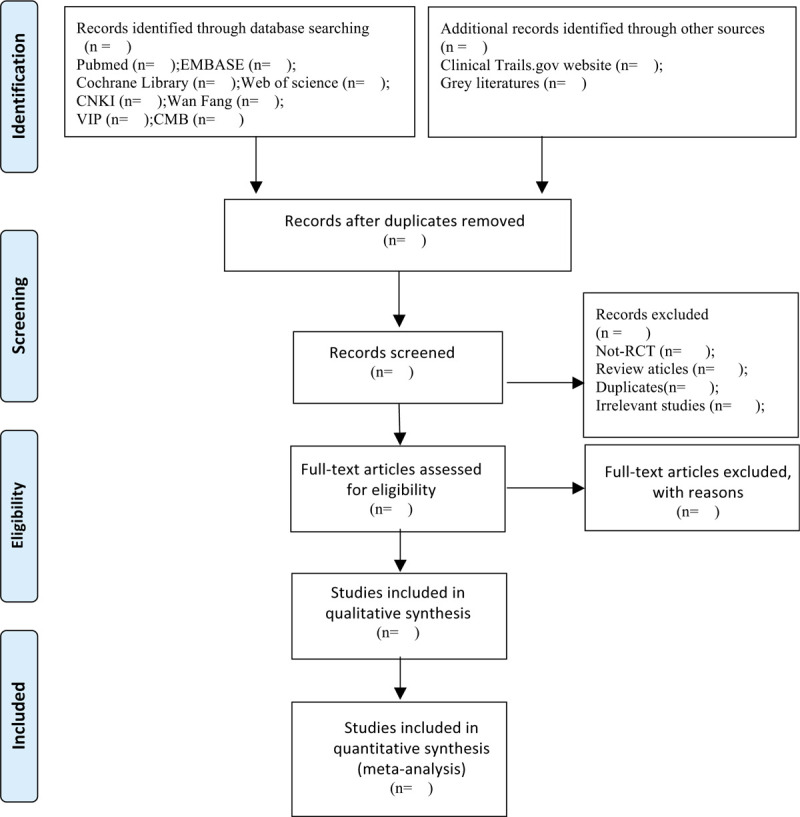
Study selection flow chart.

Researchers will import all the literatures into EndnoteX9 software for collation. In the first step, we will conduct a preliminary screening of the literature. The researchers will screen the literature that meets the inclusion criteria by reading the title and abstract. In the 2nd part, we will conduct a depth screening for the preliminary screening of the literature that meets the criteria, the researchers will browse the full text more thoughtfully and carefully to further determine whether to include or exclude. Finally, the final included literatures will be exchanged and checked by researchers with each other. If the 2 researchers disagree on the results of a study or eventual inclusion, we will resolve it through discussion, or consult with a 3rd person. Flow chart of the screening process of the study is shown in Figure 1.

#### 2.5.2 Data extraction and management

We will design an appropriate data extraction table according to the characteristics of the included study, and the data extraction process will be completed separately by 2 researchers. The 2 researchers fill in each of the raw materials included in the study according to the requirements in the data extraction form, and if any dispute occurs in the process, we will discuss it or consult with a third researcher to resolve it. The data extraction table mainly includes the following contents: research title, 1st author, year of publication, sample size (including experimental group and control group), duration of disease, intervention measures, outcome indicators (including primary and secondary results), adverse reactions, and so on. If we find that there is a lack of crucial information in the process of extraction, we can solve it by contacting the author of the article. Still, we may consider excluding the study if we cannot find the relevant information in various ways.

#### 2.5.3 Risk of bias assessment

According to the guidance of the Cochrane Handbook for Systematic Reviews of Interventions, we will conduct a bias risk assessment for inclusion in the study. The evaluation of each study mainly includes the following seven aspects: random sequence generation, allocation hiding, blinding of participants and personnel, blinding of outcome assessment, incomplete outcome data, incomplete outcome data, selective outcome reporting, and other bias. In the end, the bias of the study will be rated on 3 levels: “low bias,” “high bias,” and “ambiguous bias.”

#### 2.5.4 Measures of treatment effect

Different data types have various effect indicators. For dichotomous variables, we choose relative risk and 95% confidence interval as effect scale indicators. On the contrary, for continuous variables, it is expressed by mean difference, or standardized mean difference and 95% confidence interval. As to use mean difference or standardized mean difference, depending on the measurement scale of the data is consistent or not.

#### 2.5.5 Management of missing data

If some relevant important information is not available in the included original study, we will get in touch with the author of the article directly by e-mail or phone to obtain the information. If the missing information is still not received, then we can conduct a sensitivity analysis of the missing information to determine the impact of the lost data on the results of the whole study.

#### 2.5.6 Assessment of heterogeneity

Chi-squared test and *I*^2^ test will be applied to evaluate the heterogeneity. If the final result of the study shows that *I*^2^ < 50%, *P* > .1, then there will be no heterogeneity in these studies, and we choose the fixed effect model to synthesize the data. Conversely, *I*^2^ ≥ 50%, *P* < .1, the random effect model will be used for study, because this indicates that there is significant heterogeneity between studies. In addition, we will conduct a subgrouping or sensitivity analysis to find the source of heterogeneity.

#### 2.5.7 Assessment of reporting biases

If there are more than ten studies in this research, a funnel plot or egger test will be applied to examine publication bias, and results will be interpreted cautiously.

#### 2.5.8 Subgroup analysis

If the heterogeneity is relatively large in our study, we will be advised to conduct a subgroup analysis for different reasons. Heterogeneity may mainly come from the following several aspects: duration of intervention in study, vitamin D dosage form, dosage, etc. If a sufficient number of studies are included, we can further conduct meta-regression to explore the heterogeneity between studies.

#### 2.5.9 Sensitivity analysis

To evaluate the quality and robustness of the merger results of the whole study, a sensitivity analysis according to the recommendations of the Cochrane Handbook will be conducted by us. The method is to eliminate low-quality literature in turn and recombine the effects to evaluate the influence of the elimination of a single study on the overall results.

### 2.6 Grading the quality of evidence

For the quality evaluation of the whole study, we use the grading method of “recommended Evaluation, Development and Evaluation (Grade) Guide.” It is evaluated according to the 5 aspects of the study: limitations, inconsistencies, indirectness, inaccuracy, and publication bias of the research design. In the end, the quality of the research will be divided into 4 levels from high to low are high, medium, low, and very low.

### 2.7 Ethics and dissemination

This meta-analysis is not required for ethical approval for the data that has been published. Our findings will eventually be published in peer-reviewed journals.

## 3 Discussion

The PDN, a syndrome of chronic pain in peripheral neuropathic, is estimated that the incidence more than 20% among the diabetic population.^[16]^ It is closely related to sleep disorder, weight loss, and neuropsychiatric symptoms including anxiety and depression that affect people's quality of life. The causes of pain in DPN patients are extremely complicated. At present, the treatment of neuropathic pain is mainly symptomatic, including tricyclic antidepressants, serotonin-norepinephrine reuptake inhibitors, and calcium channel α-2-δ ligands. However, the clinical application of these drugs is often limited by unbearable adverse reactions, so the treatment of PDN patients should not be limited to a single treatment.^[8]^ Early screening and diagnosis are indispensable.

It is prevalent that vitamin D is deficient in China and around the world, especially in people with diabetes. And the deficiency of vitamin D has been considered as an independent risk element for the development of DPN.^[17]^ As we all know, vitamin D has a neuroprotective effect, which may be related to the regulation of neurotrophin level and neuronal calcium homeostasis. More and more studies have shown that vitamin deficiency may exert a greater influence on the long-term chronic complications of diabetes mellitus, especially in patients with PDN. Due to the general lack of tendency and the advantages of low side effects, vitamin D therapy is considered to be a promising intervention for the treatment of PDN.^[18]^ Therefore, the main purpose of this study is to conduct a systematic evaluation and meta-analysis of the efficacy and safety of vitamin D supplementation in the treatment of PDN, to provide some benefits for the early treatment and prevention of PDN.

## Author contributions

**Conceptualization:** Wenjing Wei, Yanli Zhang.

**Data curation:** Wenjing Wei, Rumeng Chen, Xianliang Qiu.

**Investigation:** Wenjing Wei, Rumeng Chen.

**Methodology:** Wenjing Wei, Yang Gao.

**Software:** Wenjing Wei, Yanli Zhang.

**Supervision:** Qiu Chen.

**Writing – original draft:** Wenjing Wei.

**Writing-review:** Qiu Chen, Yanli Zhang.

QC is the guarantor. All authors read, provided feedback, and approved the final manuscript.
